# (1,4,7,10-Tetra­oxacyclo­dodeca­ne)(trideuteroacetonitrile)lithium perchlorate

**DOI:** 10.1107/S1600536810009530

**Published:** 2010-03-24

**Authors:** Ilia A. Guzei, Lara C. Spencer, Lingyun Xiao, Ronald R. Burnette

**Affiliations:** a Department of Chemistry, University of Wisconsin-Madison, 1101 University Ave, Madison, WI 53706, USA; b Small Molecule Process & Product Development, AMGEN, One Amgen Center Drive, Thousand Oaks, CA 91320, USA; c School of Pharmacy, University of Wisconsin-Madison, 777 Highland Ave, Madison, WI 53705, USA

## Abstract

In the title compound, [Li(C_8_H_16_O_4_)(CD_3_CN)]ClO_4_, the Li atom is penta­coordinate. The O atoms of the 12-crown-4 ether form the basal plane, whereas the N atom of the trideutero­aceto­nitrile occupies the apical position. The Li^+^ atom is displaced by 0.794 (6) Å toward the apical position from the plane formed by the O atoms because the Li^+^ atom is too large to fit in the cavity of the 12-crown-4 ether, resulting in a distorted square-pyramidal geometry about the Li^+^ atom.

## Related literature

For applications of crown ethers, see: Jagannadh & Sarma (1999 ▶); Lehn (1973 ▶, 1995 ▶); Doyle & McCord (1998 ▶); Blasius *et al.* (1982 ▶); Blasius & Janzen (1982 ▶); Hayashita *et al.* (1992 ▶); Frühauf & Zeller (1991 ▶). For 12-crown-4 ether geometry, see: Raithby *et al.* (1997 ▶); Jones *et al.* (1997 ▶). For the size of the crown ether cavity and lithium ion, see: Shoham *et al.* (1983 ▶); Dalley (1978 ▶). For a description of the Cambridge Structural Database, see: Allen (2002 ▶). Bond distances and angles were confirmed to be typical by a *Mogul* structural check (Bruno *et al.*, 2002 ▶). For a description of tris­(1,4,7,10-tetra­oxacyclo­dodeca­ne)dilithium bis(perchlorate), synthesized simultaneously with the title compound, see: Guzei *et al.* (2010 ▶).
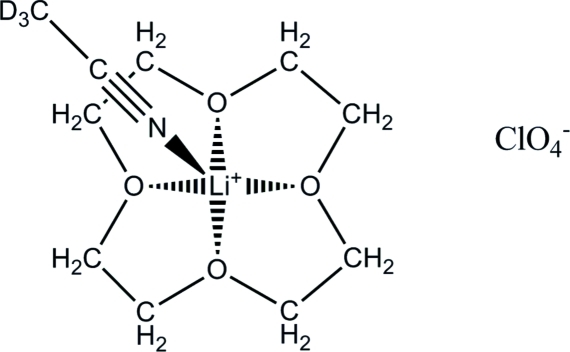


## Experimental

### 

#### Crystal data

[Li(C_8_H_16_O_4_)(C_2_D_3_N)]ClO_4_
*M* *_r_* = 323.65
Orthorhombic, 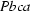

*a* = 12.1605 (14) Å
*b* = 12.6338 (15) Å
*c* = 19.870 (2) Å
*V* = 3052.7 (6) Å^3^
*Z* = 8
Mo *K*α radiation
μ = 0.29 mm^−1^
*T* = 100 K
0.40 × 0.30 × 0.20 mm

#### Data collection

Bruker CCD-1000 area-detector diffractometer
Absorption correction: multi-scan (*SADABS*; Bruker, 2003 ▶) *T*_min_ = 0.895, *T*_max_ = 0.945
20941 measured reflections
2621 independent reflections
2164 reflections with *I* > 2σ(*I*)
*R*_int_ = 0.030

#### Refinement

*R*[*F* ^2^ > 2σ(*F* ^2^)] = 0.073
*wR*(*F* ^2^) = 0.214
*S* = 1.03
2621 reflections
191 parameters
H-atom parameters constrained
Δρ_max_ = 0.81 e Å^−3^
Δρ_min_ = −0.36 e Å^−3^

### 

Data collection: *SMART* (Bruker, 2003 ▶); cell refinement: *SAINT* (Bruker, 2003 ▶); data reduction: *SAINT*; program(s) used to solve structure: *SHELXTL* (Sheldrick, 2008 ▶); program(s) used to refine structure: *SHELXTL* and *FCF_filter* (Guzei, 2007 ▶); molecular graphics: *SHELXTL* and *DIAMOND* (Brandenburg, 1999 ▶); software used to prepare material for publication: *SHELXTL*, *publCIF* (Westrip, 2010 ▶) and *modiCIFer* (Guzei, 2007 ▶).

## Supplementary Material

Crystal structure: contains datablocks global, I. DOI: 10.1107/S1600536810009530/si2247sup1.cif

Structure factors: contains datablocks I. DOI: 10.1107/S1600536810009530/si2247Isup2.hkl

Additional supplementary materials:  crystallographic information; 3D view; checkCIF report

## Figures and Tables

**Table 1:** Selected bond lengths (Å)

O1—Li1	2.022 (6)
O2—Li1	2.058 (6)
O3—Li1	2.036 (6)
O4—Li1	2.050 (6)
N1—Li1	2.010 (6)

**Fig. 1.**
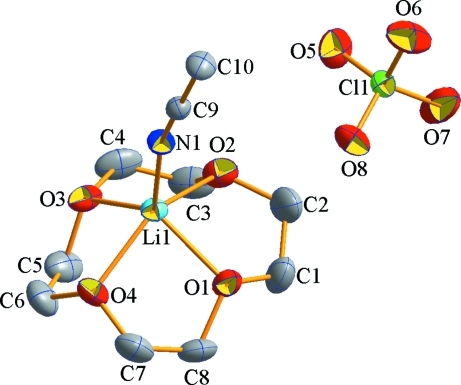
Molecular structure of (I). The thermal ellipsoids are shown at 50% probability level. All hydrogen atoms were omitted for clarity.

## supplementary crystallographic information

### Comment

Crown ethers are important due to their remarkable selectivity toward complexation with metal ions through oxygen atoms on the crown ether ring. They have high conformational flexibility, act as host molecules for various guests (Jagannadh *et al.*, 1999), and have a broad range of applications. Their importance has been studied in numerous fields such as molecular design (Lehn, 1973), supramolecular chemistry (Lehn, 1995), analytical chemistry (Doyle & McCord, 1998; Blasius *et al.*, 1982; Blasius & Janzen, 1982; Hayashita *et al.*, 1992) and medicine (Frühauf & Zeller, 1991). The ionic title compound (I), a crown ether/Li^+^ system, was prepared in a study aiming to develop a systematic methodology to understand the nature of these complexes. This methodology based on experimental crystallography may find application in characterization of host-guest type drug delivery systems.

Compound (I) crystallizes with discrete cations and anions. The Li^+^ atom of the cation exhibits a distorted square pyramidal geometry. The four oxygen atoms of the 12-crown-4 ether (12C4) bond to lithium in the basal positions, and the acetonitrile nitrogen atom occupies the apical position. The 12C4 is in the frequently observed [3333] conformation approximating C_4_ symmetry (Raithby *et al.*, 1997; Jones *et al.*, 1997). The oxygen atoms are nearly planar with a rms of 0.1325 (14) Å. The lithium atom is displaced out of this plane toward the apical position by 0.794 (6) Å. This displacement results from the Li^+^ being too large to fit in the cavity of the crown ether. The two diagonal distances across the ring between the opposite oxygens are 3.611 (4) Å and 3.890 (4) Å resulting in an adjusted diameter of the cavity between 0.811 Å and 1.090 Å. This cavity is too small to accomodate the lithium ion whose ionic diameter is between 1.18 Å and 1.52 Å (Shoham *et al.*, 1983; Dalley, 1978). The Li—N vector is nearly perpendicular to the plane of the 12C4 oxygen atoms forming a 89.8 (5)° angle. The angles and distances involving lithium were statistically similar to the averages for 14 related compounds found in the Cambridge Structural Database (CSD; Version 1.11, September 2009 release; Allen, 2002). A *Mogul* structural check also confirmed that (I) exhibits typical geometrical parameters (Bruno *et al.*, 2002).

The lithium complexes and the perchorate anions in the lattice of (I) are each stacked along a twofold screw axis to form columns along the *a* axis.

### Experimental

All chemicals were purchased from the Aldrich Chemical Co. Inc. and were used as received. 12-crown-4 (12C4, C_8_H_16_O_4_, 98% pure) and lithium perchlorate (LiClO_4_, 99% pure) were separately dissolved in acetonitrile-d_3_ (CD_3_CN, 99% pure). These two solutions were then mixed together according to a 1:1 molar ratio of 12C4/LiClO_4_.The final solution was kept in a desiccator and the solvent was allowed to evaporate gradually in order to produce a supersaturated solution. The supersaturated solution was stored at –20 °C refrigerator, until crystals formed after 48 hours. Two types of colorless crystals suitable for X-ray diffraction were obtained and separated from the solution, one of which was compound (I) the other being tris(1,4,7,10-tetraoxacyclododecane)-di-lithium diperchlorate (Guzei *et al*., 2010).

### Refinement

All H and D atoms were placed in idealized locations and refined as riding, with C—H=0.99 Å and *U*_iso_(H) = 1.2*U*_eq_(C) for all hydrogen atoms, and C—D=0.98 Å and *U*_iso_(D)=1.5*U*_eq_(C) for all deuterium atoms.

### Crystal data

**Table:**

[Li(C_8_H_16_O_4_)(C_2_D_3_N)]ClO_4_	*F*(000) = 1360
*M**_r_* = 323.65	*D*_x_ = 1.408 Mg m^−^^3^
Orthorhombic, *P**b**c**a*	Mo *K*α radiation, λ = 0.71073 Å
Hall symbol: -P 2ac 2ab	Cell parameters from 999 reflections
*a* = 12.1605 (14) Å	θ = 2.5–24.8°
*b* = 12.6338 (15) Å	µ = 0.28 mm^−^^1^
*c* = 19.870 (2) Å	*T* = 100 K
*V* = 3052.7 (6) Å^3^	Block, colourless
*Z* = 8	0.40 × 0.30 × 0.20 mm

### Data collection

**Table:**

Bruker CCD-1000 area-detector diffractometer	2621 independent reflections
Radiation source: fine-focus sealed tube	2164 reflections with *I* > 2σ(*I*)
graphite	*R*_int_ = 0.030
0.30° ω and 0.4 ° φ scans	θ_max_ = 24.8°, θ_min_ = 2.5°
Absorption correction: multi-scan (*SADABS*; Bruker, 2003)	*h* = −14→14
*T*_min_ = 0.895, *T*_max_ = 0.945	*k* = −14→14
20941 measured reflections	*l* = −23→23

### Refinement

**Table:**

Refinement on *F*^2^	Primary atom site location: structure-invariant direct methods
Least-squares matrix: full	Secondary atom site location: difference Fourier map
*R*[*F*^2^ > 2σ(*F*^2^)] = 0.073	Hydrogen site location: inferred from neighbouring sites
*wR*(*F*^2^) = 0.214	H-atom parameters constrained
*S* = 1.03	*w* = 1/[σ^2^(*F*_o_^2^) + (0.1041*P*)^2^ + 6.1747*P*] where *P* = (*F*_o_^2^ + 2*F*_c_^2^)/3
2621 reflections	(Δ/σ)_max_ = 0.024
191 parameters	Δρ_max_ = 0.81 e Å^−^^3^
0 restraints	Δρ_min_ = −0.36 e Å^−^^3^

### Special details

**Table:**

Geometry. All esds (except the esd in the dihedral angle between two l.s. planes) are estimated using the full covariance matrix. The cell esds are taken into account individually in the estimation of esds in distances, angles and torsion angles; correlations between esds in cell parameters are only used when they are defined by crystal symmetry. An approximate (isotropic) treatment of cell esds is used for estimating esds involving l.s. planes.
Refinement. Refinement of *F*^2^ against ALL reflections. The weighted *R*-factor wR and goodness of fit *S* are based on *F*^2^, conventional *R*-factors *R* are based on *F*, with *F* set to zero for negative *F*^2^. The threshold expression of *F*^2^ > σ(*F*^2^) is used only for calculating *R*-factors(gt) etc. and is not relevant to the choice of reflections for refinement. *R*-factors based on *F*^2^ are statistically about twice as large as those based on *F*, and *R*- factors based on ALL data will be even larger.

### Fractional atomic coordinates and isotropic or equivalent isotropic displacement parameters (Å^2^)

**Table:**

	*x*	*y*	*z*	*U*_iso_*/*U*_eq_	
Cl1	0.13806 (7)	0.22119 (7)	0.03636 (4)	0.0480 (3)	
O1	0.0293 (2)	0.1535 (2)	0.28852 (16)	0.0647 (8)	
O2	0.2455 (2)	0.1542 (2)	0.27072 (15)	0.0672 (8)	
O3	0.2453 (2)	−0.0130 (3)	0.35435 (13)	0.0637 (8)	
O4	0.0258 (2)	−0.0332 (2)	0.34845 (13)	0.0627 (8)	
O5	0.2322 (3)	0.1706 (3)	0.06187 (18)	0.0885 (11)	
O6	0.1349 (3)	0.2031 (4)	−0.03414 (17)	0.1030 (15)	
O7	0.1441 (3)	0.3323 (3)	0.0458 (3)	0.1151 (17)	
O8	0.0413 (3)	0.1795 (3)	0.06594 (17)	0.0792 (10)	
N1	0.1324 (2)	−0.0384 (3)	0.19108 (15)	0.0479 (8)	
Li1	0.1361 (4)	0.0312 (5)	0.2820 (3)	0.0427 (13)	
C1	0.0820 (5)	0.2547 (4)	0.2874 (3)	0.0878 (17)	
H1A	0.0332	0.3078	0.2662	0.105*	
H1B	0.0984	0.2782	0.3339	0.105*	
C2	0.1800 (5)	0.2452 (4)	0.2504 (4)	0.0945 (18)	
H2A	0.2242	0.3103	0.2563	0.113*	
H2B	0.1618	0.2387	0.2020	0.113*	
C3	0.3096 (4)	0.1645 (5)	0.3295 (3)	0.0889 (18)	
H3A	0.2669	0.2001	0.3654	0.107*	
H3B	0.3765	0.2068	0.3203	0.107*	
C4	0.3396 (4)	0.0557 (6)	0.3504 (3)	0.0899 (19)	
H4A	0.3927	0.0261	0.3176	0.108*	
H4B	0.3760	0.0584	0.3949	0.108*	
C5	0.1849 (5)	−0.0045 (5)	0.4155 (2)	0.0873 (17)	
H5A	0.2286	−0.0337	0.4532	0.105*	
H5B	0.1688	0.0707	0.4253	0.105*	
C6	0.0857 (5)	−0.0617 (5)	0.4086 (2)	0.0805 (15)	
H6A	0.0387	−0.0483	0.4484	0.097*	
H6B	0.1024	−0.1384	0.4073	0.097*	
C7	−0.0691 (4)	0.0308 (5)	0.3513 (3)	0.0782 (14)	
H7A	−0.1077	0.0182	0.3944	0.094*	
H7B	−0.1195	0.0107	0.3143	0.094*	
C8	−0.0425 (4)	0.1423 (4)	0.3456 (2)	0.0767 (14)	
H8A	−0.1103	0.1844	0.3389	0.092*	
H8B	−0.0055	0.1673	0.3870	0.092*	
C9	0.1229 (3)	−0.0579 (3)	0.13610 (17)	0.0405 (8)	
C10	0.1084 (4)	−0.0830 (4)	0.06532 (18)	0.0582 (10)	
D10A	0.0403	−0.1235	0.0594	0.087*	
D10B	0.1709	−0.1251	0.0496	0.087*	
D10C	0.1040	−0.0173	0.0392	0.087*	

### Atomic displacement parameters (Å^2^)

**Table:**

	*U*^11^	*U*^22^	*U*^33^	*U*^12^	*U*^13^	*U*^23^
Cl1	0.0511 (6)	0.0499 (6)	0.0430 (6)	−0.0013 (4)	0.0001 (4)	0.0025 (4)
O1	0.0504 (16)	0.0519 (16)	0.092 (2)	0.0035 (12)	0.0154 (15)	0.0002 (15)
O2	0.0497 (15)	0.0672 (18)	0.085 (2)	−0.0098 (13)	0.0005 (15)	−0.0171 (15)
O3	0.0570 (17)	0.089 (2)	0.0449 (14)	0.0253 (15)	−0.0082 (12)	−0.0116 (14)
O4	0.0598 (17)	0.082 (2)	0.0460 (15)	−0.0077 (15)	0.0056 (13)	0.0086 (13)
O5	0.071 (2)	0.102 (3)	0.093 (2)	0.0072 (19)	−0.0286 (19)	0.019 (2)
O6	0.089 (3)	0.175 (5)	0.0449 (19)	0.010 (3)	−0.0026 (16)	−0.001 (2)
O7	0.095 (3)	0.057 (2)	0.193 (5)	−0.0080 (19)	0.038 (3)	−0.019 (3)
O8	0.072 (2)	0.091 (2)	0.075 (2)	−0.0203 (18)	0.0178 (16)	0.0094 (18)
N1	0.0537 (18)	0.0506 (18)	0.0395 (17)	−0.0010 (13)	0.0001 (13)	−0.0062 (13)
Li1	0.044 (3)	0.049 (3)	0.034 (3)	0.002 (2)	−0.002 (2)	−0.007 (2)
C1	0.082 (4)	0.054 (3)	0.128 (5)	0.010 (3)	0.006 (3)	0.013 (3)
C2	0.103 (4)	0.060 (3)	0.121 (5)	−0.015 (3)	0.030 (4)	0.010 (3)
C3	0.059 (3)	0.114 (5)	0.093 (4)	−0.029 (3)	0.004 (3)	−0.044 (3)
C4	0.045 (2)	0.143 (6)	0.081 (3)	0.017 (3)	−0.021 (2)	−0.037 (4)
C5	0.089 (4)	0.130 (5)	0.043 (2)	0.027 (4)	−0.006 (2)	0.000 (3)
C6	0.106 (4)	0.088 (4)	0.047 (2)	−0.012 (3)	0.002 (3)	0.013 (2)
C7	0.057 (3)	0.111 (4)	0.067 (3)	−0.014 (3)	0.012 (2)	0.001 (3)
C8	0.054 (3)	0.099 (4)	0.077 (3)	0.020 (3)	0.017 (2)	−0.017 (3)
C9	0.0419 (18)	0.0396 (18)	0.0401 (19)	0.0004 (14)	−0.0002 (14)	−0.0042 (14)
C10	0.075 (3)	0.064 (2)	0.0359 (19)	0.005 (2)	−0.0047 (18)	−0.0088 (17)

### Geometric parameters (Å, °)

**Table:**

Cl1—O5	1.406 (3)	C2—H2A	0.9900
Cl1—O8	1.416 (3)	C2—H2B	0.9900
Cl1—O7	1.418 (4)	C3—C4	1.481 (9)
Cl1—O6	1.420 (4)	C3—H3A	0.9900
O1—C1	1.430 (6)	C3—H3B	0.9900
O1—C8	1.439 (5)	C4—H4A	0.9900
O1—Li1	2.022 (6)	C4—H4B	0.9900
O2—C3	1.411 (6)	C5—C6	1.413 (8)
O2—C2	1.456 (7)	C5—H5A	0.9900
O2—Li1	2.058 (6)	C5—H5B	0.9900
O3—C5	1.424 (5)	C6—H6A	0.9900
O3—C4	1.440 (7)	C6—H6B	0.9900
O3—Li1	2.036 (6)	C7—C8	1.449 (7)
O4—C7	1.411 (6)	C7—H7A	0.9900
O4—C6	1.444 (6)	C7—H7B	0.9900
O4—Li1	2.050 (6)	C8—H8A	0.9900
N1—C9	1.126 (4)	C8—H8B	0.9900
N1—Li1	2.010 (6)	C9—C10	1.452 (5)
C1—C2	1.405 (8)	C10—D10A	0.9800
C1—H1A	0.9900	C10—D10B	0.9800
C1—H1B	0.9900	C10—D10C	0.9800
			
O5—Cl1—O8	111.0 (2)	O2—C3—H3A	110.5
O5—Cl1—O7	111.1 (3)	C4—C3—H3A	110.5
O8—Cl1—O7	110.9 (2)	O2—C3—H3B	110.5
O5—Cl1—O6	107.7 (2)	C4—C3—H3B	110.5
O8—Cl1—O6	109.1 (2)	H3A—C3—H3B	108.7
O7—Cl1—O6	106.9 (3)	O3—C4—C3	112.3 (4)
C1—O1—C8	111.9 (4)	O3—C4—H4A	109.2
C1—O1—Li1	113.2 (3)	C3—C4—H4A	109.2
C8—O1—Li1	111.4 (3)	O3—C4—H4B	109.2
C3—O2—C2	117.3 (4)	C3—C4—H4B	109.2
C3—O2—Li1	109.7 (3)	H4A—C4—H4B	107.9
C2—O2—Li1	105.8 (3)	C6—C5—O3	108.6 (4)
C5—O3—C4	114.3 (4)	C6—C5—H5A	110.0
C5—O3—Li1	104.2 (3)	O3—C5—H5A	110.0
C4—O3—Li1	108.4 (3)	C6—C5—H5B	110.0
C7—O4—C6	121.5 (4)	O3—C5—H5B	110.0
C7—O4—Li1	109.4 (3)	H5A—C5—H5B	108.4
C6—O4—Li1	107.6 (3)	C5—C6—O4	112.5 (4)
C9—N1—Li1	166.0 (4)	C5—C6—H6A	109.1
N1—Li1—O1	112.2 (3)	O4—C6—H6A	109.1
N1—Li1—O3	121.9 (3)	C5—C6—H6B	109.1
O1—Li1—O3	125.7 (3)	O4—C6—H6B	109.1
N1—Li1—O4	113.0 (3)	H6A—C6—H6B	107.8
O1—Li1—O4	80.9 (2)	O4—C7—C8	111.8 (4)
O3—Li1—O4	82.1 (2)	O4—C7—H7A	109.2
N1—Li1—O2	104.3 (3)	C8—C7—H7A	109.2
O1—Li1—O2	81.1 (2)	O4—C7—H7B	109.2
O3—Li1—O2	82.1 (2)	C8—C7—H7B	109.2
O4—Li1—O2	142.4 (3)	H7A—C7—H7B	107.9
C2—C1—O1	108.1 (4)	O1—C8—C7	107.0 (4)
C2—C1—H1A	110.1	O1—C8—H8A	110.3
O1—C1—H1A	110.1	C7—C8—H8A	110.3
C2—C1—H1B	110.1	O1—C8—H8B	110.3
O1—C1—H1B	110.1	C7—C8—H8B	110.3
H1A—C1—H1B	108.4	H8A—C8—H8B	108.6
C1—C2—O2	112.8 (4)	N1—C9—C10	178.9 (4)
C1—C2—H2A	109.0	C9—C10—D10A	109.5
O2—C2—H2A	109.0	C9—C10—D10B	109.5
C1—C2—H2B	109.0	D10A—C10—D10B	109.5
O2—C2—H2B	109.0	C9—C10—D10C	109.5
H2A—C2—H2B	107.8	D10A—C10—D10C	109.5
O2—C3—C4	106.3 (4)	D10B—C10—D10C	109.5
			
C9—N1—Li1—O1	25.1 (15)	C2—O2—Li1—N1	91.0 (4)
C9—N1—Li1—O3	−150.4 (12)	C3—O2—Li1—O1	107.7 (3)
C9—N1—Li1—O4	114.5 (13)	C2—O2—Li1—O1	−19.7 (4)
C9—N1—Li1—O2	−60.9 (14)	C3—O2—Li1—O3	−20.5 (4)
C1—O1—Li1—N1	−106.3 (4)	C2—O2—Li1—O3	−147.9 (3)
C8—O1—Li1—N1	126.6 (4)	C3—O2—Li1—O4	45.4 (6)
C1—O1—Li1—O3	69.1 (5)	C2—O2—Li1—O4	−82.0 (6)
C8—O1—Li1—O3	−58.0 (5)	C8—O1—C1—C2	155.6 (5)
C1—O1—Li1—O4	142.5 (4)	Li1—O1—C1—C2	28.7 (6)
C8—O1—Li1—O4	15.4 (3)	O1—C1—C2—O2	−48.1 (7)
C1—O1—Li1—O2	−4.4 (4)	C3—O2—C2—C1	−79.4 (6)
C8—O1—Li1—O2	−131.5 (3)	Li1—O2—C2—C1	43.3 (6)
C5—O3—Li1—N1	−143.2 (4)	C2—O2—C3—C4	163.1 (4)
C4—O3—Li1—N1	94.7 (4)	Li1—O2—C3—C4	42.5 (5)
C5—O3—Li1—O1	41.8 (5)	C5—O3—C4—C3	−82.2 (5)
C4—O3—Li1—O1	−80.3 (4)	Li1—O3—C4—C3	33.5 (5)
C5—O3—Li1—O4	−31.0 (4)	O2—C3—C4—O3	−51.2 (6)
C4—O3—Li1—O4	−153.1 (3)	C4—O3—C5—C6	170.3 (5)
C5—O3—Li1—O2	114.8 (4)	Li1—O3—C5—C6	52.2 (5)
C4—O3—Li1—O2	−7.3 (3)	O3—C5—C6—O4	−51.1 (6)
C7—O4—Li1—N1	−99.2 (4)	C7—O4—C6—C5	−104.8 (5)
C6—O4—Li1—N1	126.9 (4)	Li1—O4—C6—C5	22.3 (6)
C7—O4—Li1—O1	11.2 (3)	C6—O4—C7—C8	90.0 (5)
C6—O4—Li1—O1	−122.7 (3)	Li1—O4—C7—C8	−36.3 (5)
C7—O4—Li1—O3	139.4 (3)	C1—O1—C8—C7	−165.9 (4)
C6—O4—Li1—O3	5.5 (4)	Li1—O1—C8—C7	−38.0 (5)
C7—O4—Li1—O2	73.5 (6)	O4—C7—C8—O1	49.2 (5)
C6—O4—Li1—O2	−60.4 (6)	Li1—N1—C9—C10	−72 (20)
C3—O2—Li1—N1	−141.5 (4)		
